# Preliminary Examinations of the Teeth

**Published:** 1903-04-15

**Authors:** E. A. Bogue

**Affiliations:** New York


### PRELIMINARY EXAMINATIONS OF THE TEETH.

BY E. A. BOGUE, M.D., NEW YORK.

I have been asked to explain my method of making preliminary examinations when a patient presents for treatment. I might follow the example of my revered friend, Dr. Bronson, and say it consisted of the mental isolation of each tooth from all the rest and a careful searching of every side of each tooth, and the end as well, for decayed or defective spots. These decayed or defective spots are more apt to be found in the sulci or between the teeth than elsewhere, and next to those two localities they are apt to be found at the margins of the gum.

Holding essentially to the views promulgated by Dr. Black, that all normal teeth are susceptible of being preserved through life, it behooves us to discover and mechanically correct at the earliest possible moment defects in the enamel coating of all teeth intrusted to our care. As many of the patients who come into our hands for the first time may have reached or nearly reached maturity, we may expect to find between the teeth evidences of disintegration of enamel, if not of decay of the dentin. In order to detect this disintegration at an early stage, it is necessary to have the surfaces of the tooth as they are examined quite dry, that the difference in color between sound enamel and disintegrated or chalky enamel may be perceived.

There has been placed at our disposal lately a means of easily keeping these teeth dry, not only during examinations but oftentimes for the filling of certain cavities. That means is the so-called "Aseptic Cotton Rolls," that in the first place bore the motto, "Made in Germany;" afterward they began to be made in France, and, to-day, I understand that those made in the United States are being exported both to France and Germany. I find these rolls excellent for several purposes. The first, for examinations, has already been mentioned, and I use them by laying them along both buccal and lingual sides of the lower teeth, and, holding the one below the tongue firmly down, I ask the patient to raise the tongue until the roll shall have found its way underneath it. In this manner the roll can often be made to retain its position practically during the entire examination of one side of the mouth below. Should difficulty be found in holding the roll on the lingual side in place, an addition to it can be made in the shape of a napkin folded into a long, slim triangle, which may be laid over the cotton roll and held in place either by the mouth mirror in the hand of the operator or by the index finger of the patient, or in cases of great difficulty it can be held by a Hawes napkin-holder, an instrument invented forty or fifty years ago but which serves its purpose as well to-day as ever.

Another roll being laid upon the buccal side of the teeth, below, and a third one over the mouth of Steno's duct, above, gives us an excellent opportunity to examine with dryness and with care the inter-proximal spaces by wiping them out with dry dental floss and looking at them in the mouth mirror as well as by feeling them with the fine explorers with which we make our examinations.

In arranging to examine the upper teeth the matter is less complicated. One little roll between the cheek and the teeth, against the duct of Steno, is often enough to insure dryness sufficiently long to complete an examination on one side above. Should dryness not be attained with one, a second roll can be laid forward from the first, reaching perhaps as far as the incisor teeth and then wiping between the teeth with dental floss. These rolls can be held in place in case of need either by an ordinary clamp clasping the last molar in the mouth, or by one of the clamps made by Ivory that has long projecting points, so that they will hold the rolls and napkin in place for a long time. I find, by the way, that these rolls, in conjunction with napkins folded as mentioned above, can be, by these Ivory clamps, held in place long enough to insert many of the grinding surface fillings, as well as nearly all of the gutta-percha or cement fillings and many amalgam ones as well.

These rolls have also been of wonderful service in keeping the teeth dry for the attachment of rings and regulating fixtures and for procuring dryness in a hundred other odd moments in which we want temporary dryness with the least possible delay.—*Dental Brief.*

